# The Bishop of London's Urgent Appeal on Behalf of His Diocesan Boys' Camps

**Published:** 1904-08-13

**Authors:** A. F. London

**Affiliations:** London Diocesan Seaside Camp for Working Lads, Bexhill


### THE BISHOP OF LONDON'S URGENT APPEAL ON BEHALF OF HIS DIOCESAN BOYS' CAMPS.

*To the Editor of* The Hospital.

Sir,—I hope that the numerous appeals which I have been obliged to address to the public this summer will not prejudice the one which I now make on behalf of our London boys. I am writing this surrounded by 300 happy boys, some bathing, some playing cricket, others acting as sentry and guarding the camp, and all trained and invigorated by a week at the seaside and the healthy activities of camp life. These boys return to-morrow morning, to be succeeded by 600 more, who will arrive for their week in the afternoon. It would be impossible to imagine a more ideal holiday for boys after the confinement and monotony of their work in London, but, although the boys all pay, according to their wages, towards their outing, a large sum is required for tents, the railway fares, and the catering for so large a number, and for the first time for 15 years we are face to face with a serious deficit. I hope that I shall not appeal in vain for help for so successful and necessary a work.

Cheques should be sent to Mr. F. Abel Bloxam, 23 Northumberland Avenue, Charing Cross.

Yours faithfully,
(Signed) A. F. London.

London Diocesan Seaside Camp for Working Lads,

